# The Construction and Analysis of Tumor-Infiltrating Immune Cells and ceRNA Networks in Bladder Cancer

**DOI:** 10.3389/fgene.2020.605767

**Published:** 2020-12-18

**Authors:** Aimin Jiang, Na Liu, Shuheng Bai, Jingjing Wang, Huan Gao, Xiaoqiang Zheng, Xiao Fu, Mengdi Ren, Xiaoni Zhang, Tao Tian, Zhiping Ruan, Yu Yao, Xuan Liang

**Affiliations:** ^1^Department of Medical Oncology, The First Affiliated Hospital of Xi’an Jiaotong University, Xi’an, China; ^2^Department of Radiotherapy Oncology, The First Affiliated Hospital of Xi’an Jiaotong University, Xi’an, China

**Keywords:** bladder cancer (BLCA), immune cells, ceRNA, prognosis, TCGA

## Abstract

**Background:**

Bladder cancer (BLCA) is the 11th most common malignancy worldwide. Although significant improvements have been made in screening, diagnosis, and precise management in recent years, the prognosis of BLCA remains bleak.

**Objectives:**

This study aimed to investigate the prognostic significance of tumor-infiltrating immune cells and construct ceRNA networks in BLCA patients.

**Methods:**

The expression data of BLCA patients were obtained from The Cancer Genome Atlas (TCGA) database. A competing endogenous RNA (ceRNA) network was constructed to identify the hub genes involved in the prognosis of BLCA. The CIBERSORT algorithm was utilized to investigate the infiltration levels of 22 subsets of immune cells. Ultimately, the nomogram was generated to visualize the survival probability of each patient, with the calibration curve being performed to assess its performance. Furthermore, the Pearson correlation test was used to explore the correlation between the identified hub genes in the ceRNA network and the prognostic-related immune cells.

**Results:**

A total of eight elements in the ceRNA network were considered as key members and correlated with the prognosis of BLCA, including *ELN*, *SREBF1*, *DSC2*, *TTLL7*, *DIP2C*, *SATB1*, *hsa-miR-20a-5p*, and *hsa-miR-29c-3p*. T cells CD8, T cells follicular helper (Tfh), and neutrophils were identified as independent prognostic factors in BLCA. The co-expression analysis showed that there was a significant correlation between the identified hub genes and immune cells.

**Conclusion:**

Our results suggest that the mechanism of *hsa-miR-29c-3p* regulates the expression of *ELN* and *DSC2*, and the infiltration of Tfh and neutrophils might play pivotal roles in the progression of BLCA.

## Introduction

Bladder cancer (BLCA) is the 11th most common malignancy worldwide (Babjuk et al., 2017). It is reported that approximately 900,000 new cases of BLCA are diagnosed each year, and the mortality of BLCA is staggering at 20% (Klotz and Brausi, 2015). The global BLCA age- and death- standardized incidence rates per 100,000 have been reported 6.71 and 2.96, respectively (Babjuk et al., 2017). As the most predominant pathological subtype of BLCA, urothelial cell carcinoma (UCC) accounting for more than 90% of all BLCA patients (Sharifi et al., 2019). Besides, 75% of UCC patients are classified as non-muscle-invasive bladder cancer (NMIBC), whereas the rest are muscle-invasive or metastatic disease. Although significant improvements have been made in screening, diagnosis, and precise management in recent years, the prognosis of BLCA remains bleak. Therefore, BLCA is a very significant public health problem, and it has a significant impact on mortality, quality of life, and economic cost (Klotz and Brausi, 2015).

In recent years, the clinical application of immune checkpoint inhibitors (ICIs) has demonstrated promising response rates in BLCA, approving that it could play an antitumor role by reversing immunodeficiency and activating the immune cells (Massari et al., 2018). Tumor microenvironment (TME) encompasses both cellular and non-cellular milieus that work together in tumor development, progression, and metastasis (Hanahan and Weinberg, 2011; Gajewski et al., 2013). As recent studies suggested, the infiltration levels of immune cells in the TME were involved in the antitumor immune responses. Recently, increasing evidence suggests that the competing endogenous RNA (ceRNA) networks, which are composed of long non-coding RNAs (lncRNAs), messenger RNAs (mRNAs), and microRNAs (miRNAs), regulating the crosstalk between the tumor cells and immune cells (Salmena et al., 2011). In the ceRNA networks, the many-to-one and one-to-many regulatory relationships between miRNAs, target mRNAs, and transcription factors affecting tumor biological processes via participating in gene regulation (Huang et al., 2019a). Furthermore, mounting studies revealed that the evaluation of immune cell infiltration plays a pivotal role in predicting the prognosis in various malignancies (Geiger et al., 2000; Galon et al., 2006). Increasing studies have elucidated that the ceRNA networks can predict the prognosis of BLCA patients (Wang et al., 2016; Zhu et al., 2018; Lyu et al., 2019; Jiang et al., 2020). However, no combined networks have been constructed to predict the clinical outcomes of these individuals. Therefore, we conducted the present bioinformatic analysis to investigate the prognostic significance of immune cells and construct ceRNA networks in these individuals using The Cancer Genome Atlas (TCGA) dataset.

## Materials and Methods

### Raw Data Acquisition and Analysis

The gene expression profiles of 433 BLCA patients (tumor samples, 414 cases; normal samples, 19 cases) were downloaded from the TCGA database^1^), including lncRNA, mRNA, and miRNA. Both HTseq-count and fragments per kilobase of exon per million reads mapped (FPKM) profiles were obtained. Besides, the corresponding clinical profiles of each patient were also obtained, with the Practical Extraction and Report Language (Perl) script being used to merge all the clinical data. After filtering non-BLCA specific expression genes which were not detected in either the experimental group or the control group, the ‘DEseq2’ package was exploited to identify the differentially expressed lncRNAs, mRNAs, and miRNAs. With the | logFC (fold change)| > 1.0, false discovery rate (FDR) adjusted *P*-value < 0.05 was used as filtering criteria.

### Construction of a ceRNA Network

A ceRNA network was constructed with the following steps: (a) the differentially expressed lncRNAs and overlapped with the differentially expressed miRNAs were matched through starBase^2^ (Li et al., 2014) and used for the establishment of the lncRNA/miRNA interaction; (b) miRNAs regulated for both lncRNAs and mRNAs, showing significant results (*P*-value < 0.05) in hypergeometric testing and correlation analysis, were selected to construct ceRNA network; (c) the visualization of the ceRNA network was achieved using Cytoscape version 3.8.0.

### Survival Analysis and Nomograms of Hub Genes in the ceRNA Network

The Kaplan–Meier survival curves of the members in the ceRNA network were generated to identify potential hub genes. A *P*-value < 0.05 was considered with a statistical difference. Subsequently, the Cox regression analysis and lasso regression were performed to identify optimal differentially expressed genes (DEGs) and to ensure that the multifactor models were not overfitting. Besides, the risk score of each patient was also calculated according to the results of multivariate Cox regression analysis based on the following formula:

$$\begin{array}{c} \mathrm{Survival}\,\mathrm{risk}\,\mathrm{score}\,\left(\mathrm{patient}\right)=\sum_{i=1}^{k}\mathrm{coefficient}\\ \left(\mathrm{gene}\,i\right)\,\mathrm{expression}\,\mathrm{value}\,\mathrm{of}\,\left(\mathrm{gene}\,i\right) \end{array}$$

In the formula, ‘k’ represents the total number of the DEGs in the prognostic model. ‘gene i’ represents the i^*th*^ selected DEG, and ‘coefficient (gene i)’ represents the coefficient of the DEG in multivariate Cox regression analysis. Ultimately, a nomogram was formulated based on the results of multivariate analysis to visualize the risk score of each patient. Meanwhile, the receiver operating characteristic curves (ROC) and calibration curves were also generated to assess the performance of the nomogram.

### Immune Cell Infiltration and Co-expression Analysis

The R software package, ‘CIBERSORT’ was adopted to calculate the abundance of 22 leukocyte subtypes in 414 tumor samples, and a threshold of *P*-value < 0.05 was considered as cut-off criteria. Furthermore, Wilcoxon rank-sum test was performed to analyze the difference of immune cells infiltration in BLCA and normal bladder tissues, with the R software package, ‘vioplot’ was used to visualize the difference. The Kaplan–Meier survival curves of different subtypes of immune cells were exploited to compare their survival difference in BLCA. A *P*-value < 0.05 was considered with a statistical difference. Finally, the Cox regression analysis and Lasso regression analysis were performed to find the significant immune cells which were correlated with the prognosis of BLCA, with a nomogram being used to calculate the survival probability of these individuals. Furthermore, we performed the co-expression analysis via the Pearson correlation test to analyze the relationship of hub genes in the ceRNA network and the identified prognostic-related immune cells.

### Multidimensional Validation

To minimize bias, we conducted a multidimensional validation for the identified optimal members of the ceRNA network in multiple external databases, including Oncomine^3^, the Gene Expression Profiling Interactive Analysis (GEPIA^4^), database, Kaplan–Meier plotter database^5^, UALCAN^6^, Human Protein Atlas database (HPA^7^), and Tumor IMmune Estimation Resource (TIMER^8^). Furthermore, TargetScan database^9^ was also adopted to predict the binding sites of the identified targeted genes.

### Statistical Analysis

All statistical analysis was performed using R Studio (version 1.3.959) and Bioconductor^10^. The R software package, limma, was used to differential analysis. The ‘survival’ package was adopted to the Cox regression model construction. The survival difference was evaluated and visualized using Kaplan–Meier survival curves, and the association was tested via log-rank tests. The ROC curve was adopted to assess the predictive ability of the prognostic model, with an AUC value > 0.60 was considered as acceptable for predictions, and an AUC > 0.75 was regarded as has the excellent predictive ability (Han et al., 2018; Cho et al., 2019). Only the two-sided *P*-value < 0.05 was considered to be of statistical significance.

## Results

### Screening of Differentially Expressed Genes of BLCA in TCGA Database

**FIGURE 1:**
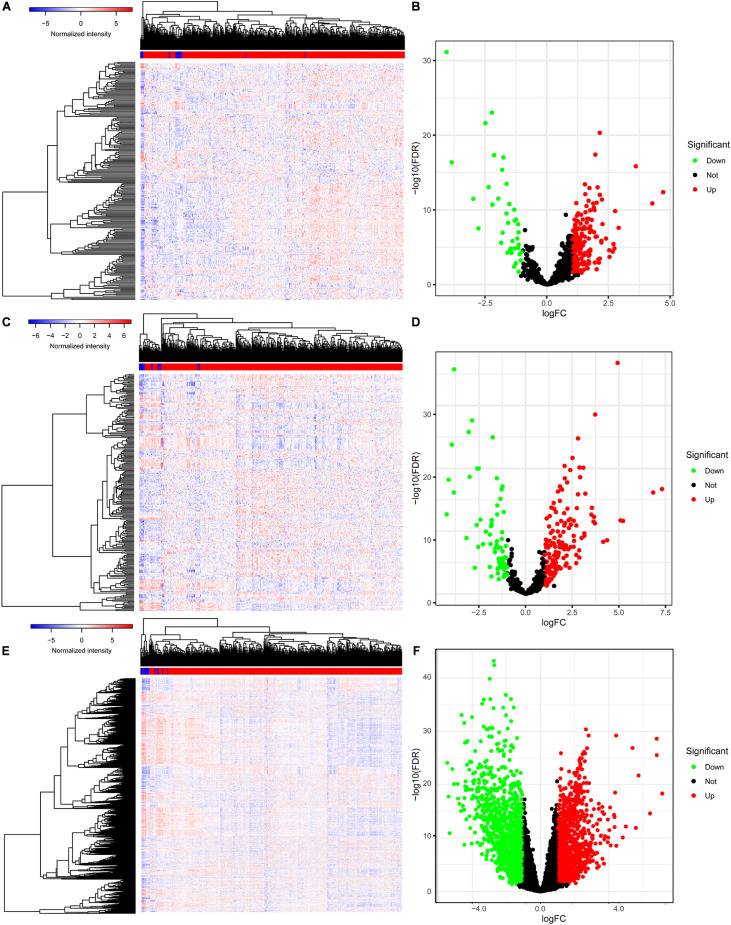
The differentially expressed genes in BLCA. The heatmap **(A)** and the volcano plot **(B)** of all differentially expressed miRNAs; the heatmap **(C)** and the volcano plot **(D)** of all differentially expressed lncRNAs; the heatmap **(E)** and the volcano plot **(F)** of all differentially expressed mRNAs. BLCA, bladder cancer; miRNAs, microRNAs; lncRNAs, long non-coding RNAs; mRNAs, messenger RNAs.

The baseline characteristics of all the patients available from the TCGA are described in Supplementary Table 1. A total of 63,071 RNAs were identified from the TCGA-BLCA cohort. Among them, there were 223 differentially expressed miRNAs (152 upregulated and 71 downregulated) (Figures 1A,B), 216 differentially expressed lncRNAs (177 upregulated and 39 downregulated) (Figures 1C,D), and 2,581 differentially expressed mRNAs (1,261 upregulated and 1,320 downregulated) (Figures 1E,F).

### Construction of ceRNA Network and Survival Analysis

**FIGURE 2:**
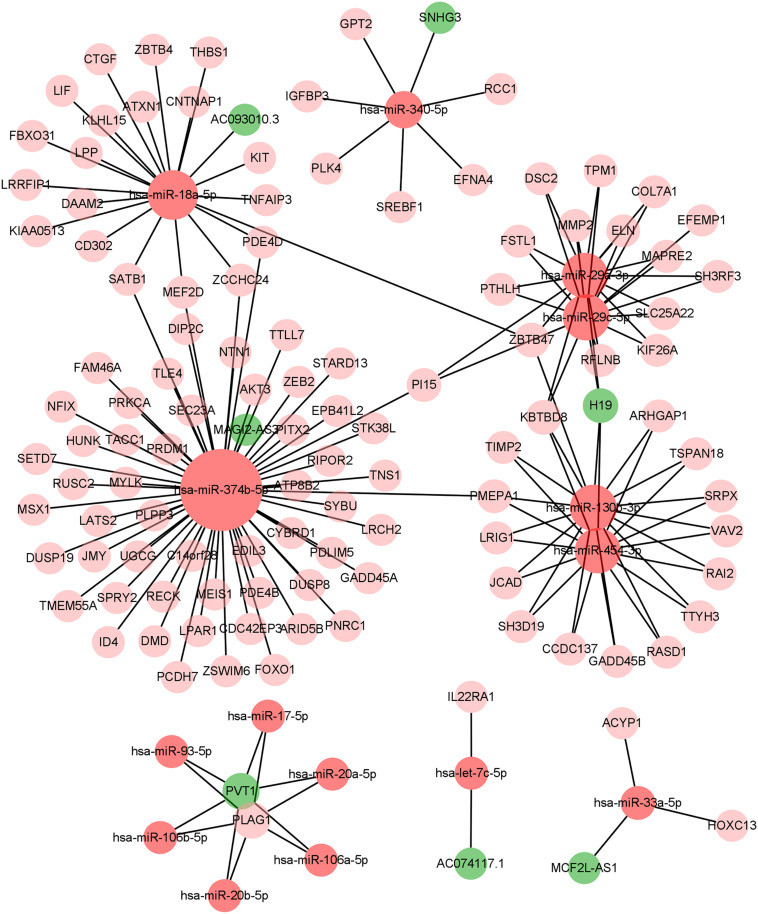
The constructed ceRNA networks via Cytoscape. ceRNA, competing endogenous RNA.

**FIGURE 3:**
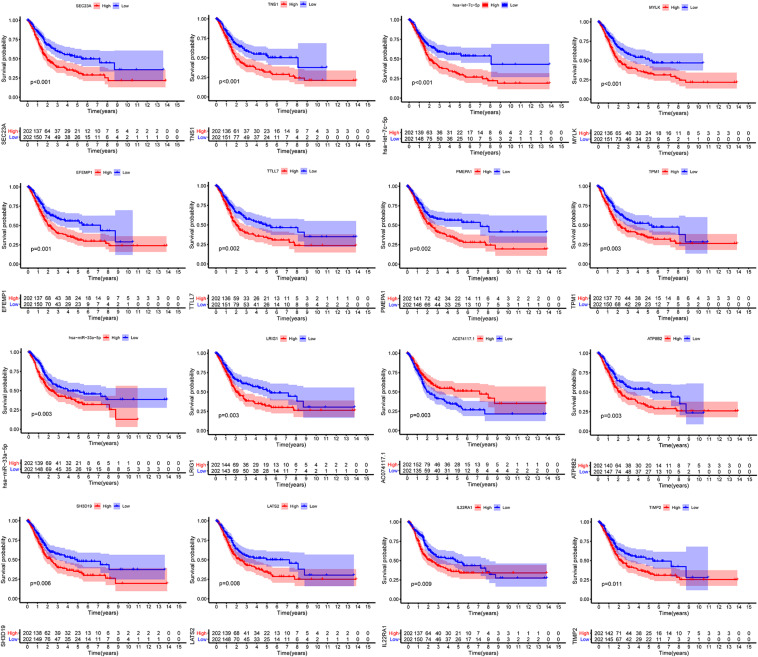
Kaplan–Meier survival curves of the former 16 genes in the ceRNA networks. ceRNA, competing endogenous RNA.

**FIGURE 4:**
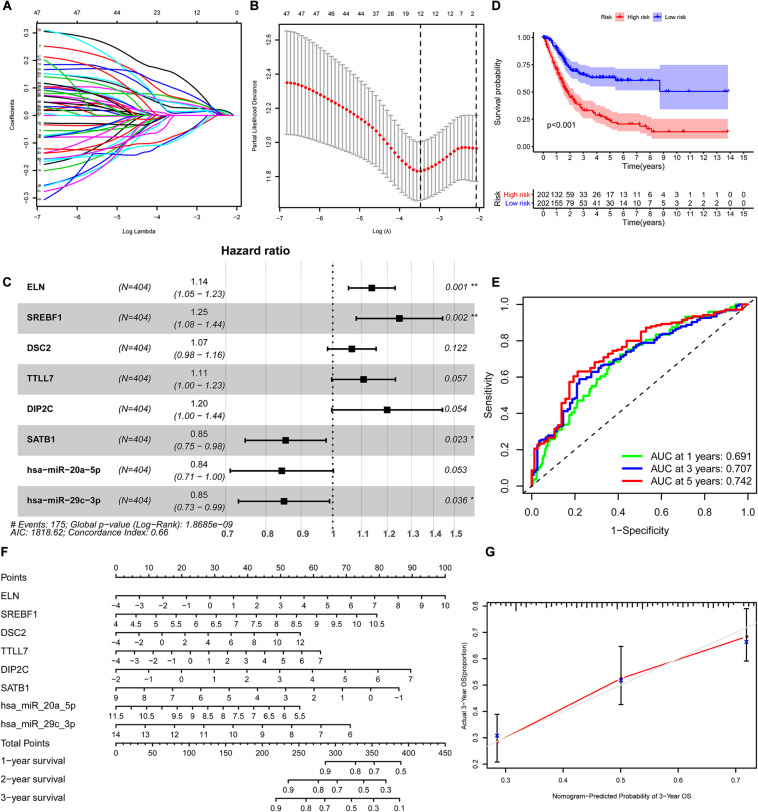
Construction of the nomogram for predicting the survival probability of BLCA based on the identified hub genes in ceRNA networks. The results of the Lasso regression **(A,B)**; the forest plot of the multivariate Cox regression analysis **(C)**; the Kaplan–Meier survival curve in high- and low- risk groups based on the multivariate Cox regression analysis **(D)**; the ROC curves of the multivariate Cox model **(E)**; the nomogram **(F)** and its calibration curve **(G)**. BLCA, bladder cancer; ceRNA, competing endogenous RNA; ROC, Receiver Operating Curve.

We constructed the ceRNA network, which was composed of 7 lncRNAs, 15 miRNAs, and 110 mRNAs based on the interactions of 15 lncRNA-miRNA pairs and 154 miRNA-mRNA pairs (Figure 2 and Supplementary Table 2). Further analysis revealed that the network contained 132 nodes and 169 edges. The Kaplan–Meier survival curves and log-rank tests were exploited to compare the survival difference of the members in the network. It showed that a total of 38 genes were significantly associated with the prognosis of BLCA, and we presented the Kaplan–Meier survival curves of the former 16 genes according to the rank of *P*-value (Figure 3). The Cox regression analysis and Lasso regression analysis were further conducted to identify the hub genes in the network. A total of 12 genes were integrated into the multivariate Cox regression analysis (Figures 4A,B). Finally, eight hub genes in the ceRNA network were considered as key members and correlated with the prognosis of BLCA through multivariate analysis, including *ELN*, *SREBF1*, *DSC2*, *TTLL7*, *DIP2C*, *SATB1*, *hsa-miR-20a-5p*, and *hsa-miR-29c-3p* (Figure 4C and Table 1). Furthermore, we classified the patients into high-risk and low-risk score groups according to the median value of risk score to determine the difference between the two groups. We then performed a Kaplan–Meier curve based on the log-rank test, indicating that patients in the high-risk score group had a worse prognosis (Figure 4D). Meanwhile, the ROC curves were generated to evaluate the predictive ability of the Cox regression hazard model, which suggested an acceptable accuracy (AUC of 1-year survival: 0.691, AUC of 3-year survival: 0.707, and AUC of 5-year survival: 0.742) (Figure 4E). Then, the nomogram was generated to depict the survival probability of each patient, with the calibration curve being performed to assess the performance of the nomogram (Figures 4F,G).

**TABLE 1:** Multivariate Cox proportional hazards regression model including the key members of the ceRNA network for overall survival in patients with BLCA.

Gene	Coefficient	HR	95%CI		*P*
			Lower	Upper	
ELN	0.13	1.14	1.05	1.23	0.001
SREBF1	0.22	1.25	1.08	1.44	0.002
SC2	0.06	1.07	0.98	1.16	0.122
TTLL7	0.10	1.11	1.00	1.23	0.057
DIP2C	0.18	1.20	1.00	1.44	0.054
SATB1	–0.16	0.85	0.75	0.98	0.023
hsa-miR-20a-5p	–0.17	0.84	0.71	1.00	0.053
hsa-miR-29c-3p	–0.16	0.85	0.73	0.99	0.036

*BLCA, bladder cancer; HR, hazard ratio; CI, confidence interval.*

### Immune Cell Infiltration and Survival Analysis in BLCA

**FIGURE 5:**
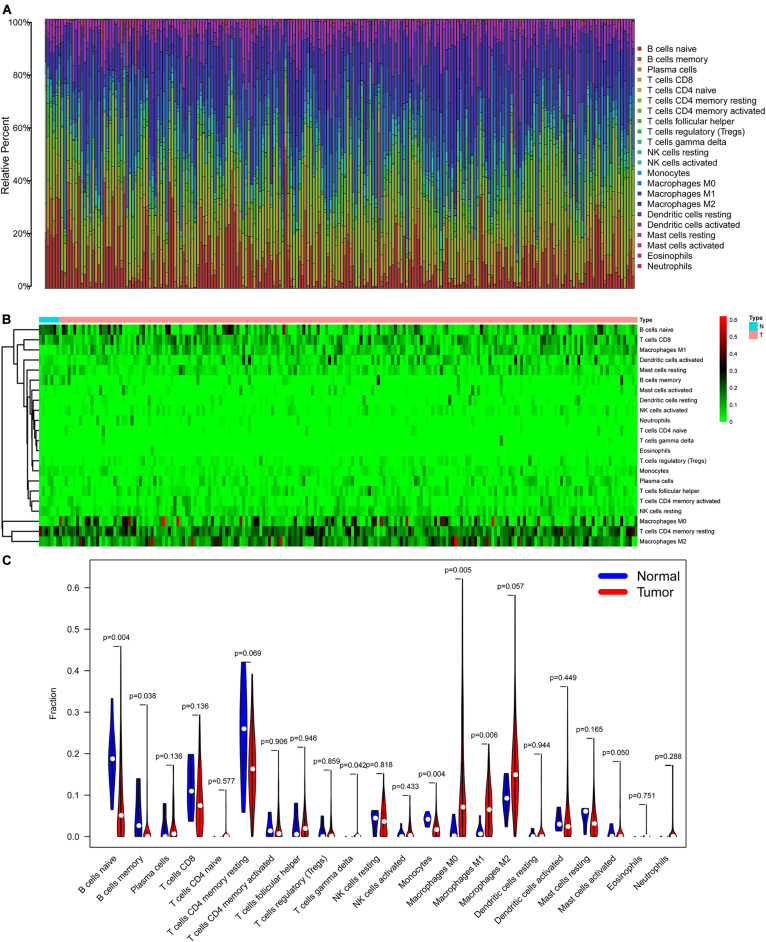
The composition **(A)** and heatmap **(B)** of 22 subsets of immune cells in BLCA; the violin plot **(C)** of immune cells infiltration in tumor and normal groups. BLCA, bladder cancer.

**FIGURE 6:**
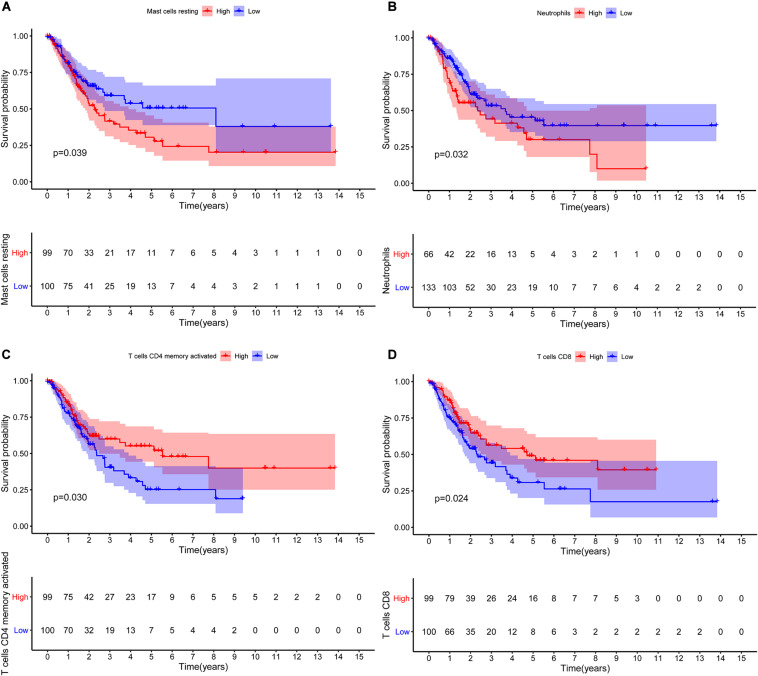
Kaplan–Meier survival curves of the prognostic-related immune cells in BLCA. Mast cells resting **(A)**; Neutrophils **(B)**; T cells CD4 memory activated **(C)**; T cells CD8 **(D)**. BLCA, bladder cancer.

**FIGURE 7:**
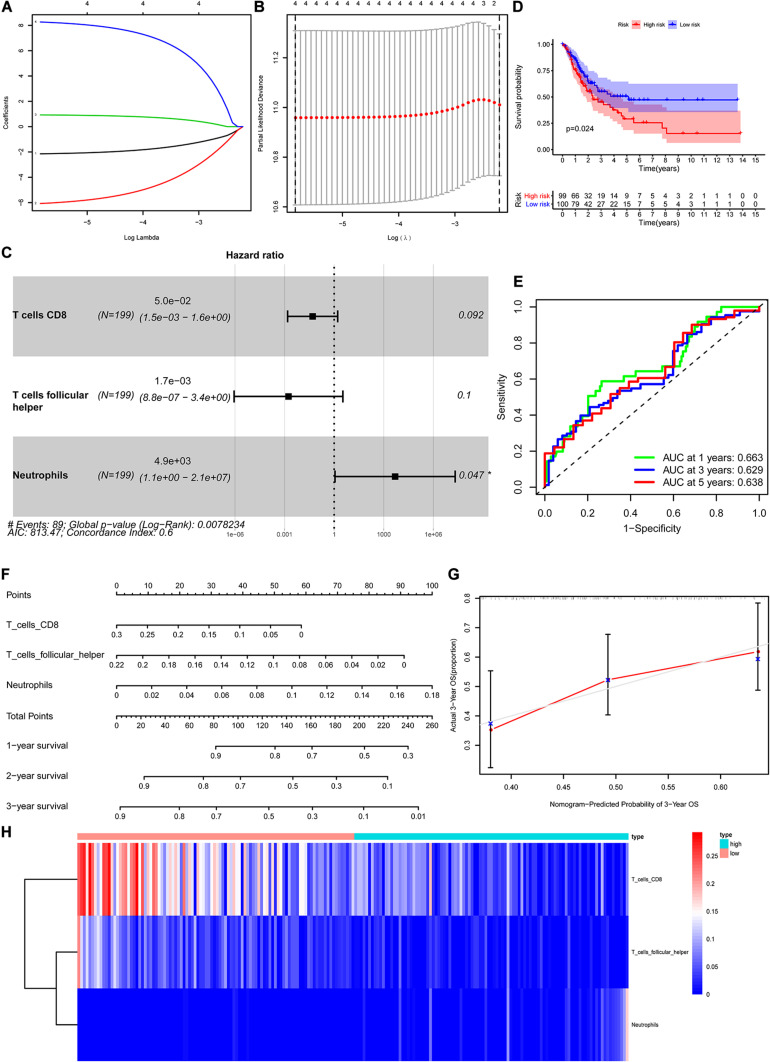
Construction of the nomogram for predicting the survival probability of BLCA based on the prognostic-related immune cells. The results of the Lasso regression **(A,B)**; the forest plot of the multivariate Cox regression analysis **(C)**; the Kaplan–Meier survival curve in high- and low- risk groups based on the multivariate Cox regression analysis **(D)**; the ROC curves of the multivariate Cox model **(E)**; the nomogram **(F)** and its calibration curve **(G)**; composition of the identified key immune cells in high- and low- risk groups **(H)**. BLCA, bladder cancer; ROC, receiver operating curve.

The CIBERSORT algorithm was used to estimate the abundance of 22 subtypes of immune cells in BLCA patients, and a threshold of *P*-value < 0.05 was considered as statistically significant. The infiltration levels of 22 subsets of immune cells were presented in Figures 5A,B. We also compared the distribution difference of these immune cells between BLCA samples and normal bladder samples. The results depicted that T cells gamma delta, Macrophages M0, and Macrophages M1 were significantly infiltrated in BLCA samples, while B cells naïve, B cells memory, and Monocytes were significantly infiltrated in normal bladder samples (Figure 5C). Then, Kaplan–Meier survival curves were plotted to find potential prognostic-related immune cells in BLCA, which suggested that the infiltration levels of Mast cells resting, Neutrophils, T cells CD4 memory activated, and T cells CD8 were significantly correlated with the prognosis of BLCA (Figures 6A–D). Subsequently, all immune cells were integrated into univariate Cox regression analysis. After the selection of Lasso regression analysis (Figures 7A,B), T cells CD8, T cells follicular helper (Tfh), and neutrophils were referred to as independent prognostic factors in BLCA (Figure 7C and Table 2). Further risk survival curve based on the multivariate analysis revealed that patients in the high-risk score group had a worse prognosis (Figure 7D). Meanwhile, the ROC curves suggested that the Cox hazard model had an acceptable accuracy in predicting the survival of BLCA (AUC of 1-year survival: 0.663, AUC of 3-year survival: 0.629, and AUC of 5-year survival: 0.638) (Figure 7E). Then, the nomogram was generated to depict the survival probability of each patient, with a calibration curve being performed to assess the performance (Figures 7F,G). Figure 7H showed the infiltration levels of T cells CD8, Tfh, and neutrophils in the low-risk score and high-risk score groups.

**TABLE 2:** Multivariate Cox proportional hazards regression model including the key immune cells for overall survival in patients with BLCA.

Immune cells	Coefficient	HR	95%CI		*P*
			Lower	Upper	
T cells CD8	–3.00	0.05	0.00	1.63	0.092
T cells follicular helper	–6.36	0.00	0.00	3.35	0.100
Neutrophils	8.49	4.86E+03	1.13	2.10E+07	0.047

*BLCA, bladder cancer; HR, hazard ratio; CI, confidence interval.*

### Co-expression Analysis

**FIGURE 8:**
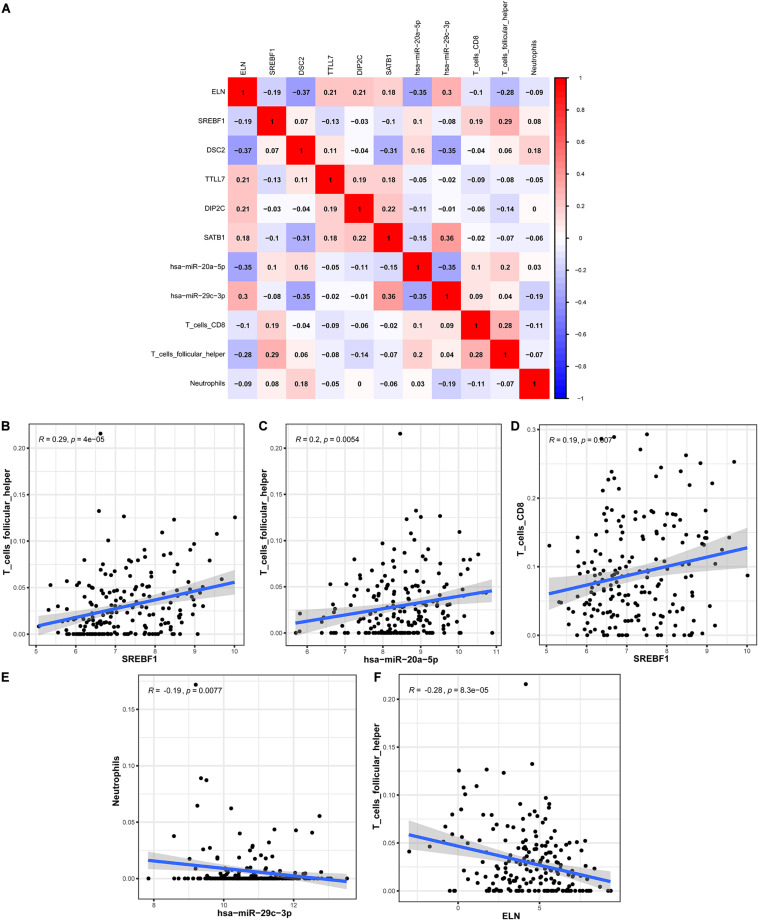
The co-expression analysis between the identified hub genes and immune cells in BLCA. The co-expression patterns among fractions of three immune cells and eight hub genes in the ceRNA network **(A)**; Linear relationship between immune cells and genes with high col-linearity **(B–F)**. BLCA, bladder cancer; ceRNA, competing endogenous RNA.

In order to explore the correlation between the identified hub genes in the ceRNA network and the prognostic-related immune cells, we performed the co-expression analysis via the Pearson correlation test. The co-expression analysis showed that there was a significant correlation between *hsa-miR-20a-5p* and *ELN*, *hsa-miR-20a-5p* and Tfh, *hsa-miR-29c-3p* and *DSC2*, *hsa-miR-29c-3p* and neutrophils (Figure 8A). Further co-expression analysis also revealed that there was a significantly positive correlation between *SREBF1* and Tfh (Figure 8B), *hsa-miR-20a-5p* and Tfh (Figure 8C), *SREBF1* and T cells CD8 (Figure 8D). Simultaneously, there was a significantly negative correlation between *hsa-miR-29c-3p* and neutrophils (Figure 8E), *ELN* and Tfh (Figure 8F).

### Multidimensional Validation

We performed a multidimensional validation in multiple external databases, including Oncomine, GEPIA, UALCAN, HPA, Kaplan–Meier Plotter, and TIMER databases, to minimize the bias. In the Oncomine database, there was no significant difference in *ELN* between BLCA samples and normal bladder samples (Median rank 2,988.5, *P* = 0.384, Supplementary Figures 1A,B). Nevertheless, we observed that the expression level of *DSC2* was apparently high in BLCA samples than normal bladder samples (Median rank 367.0, *P* = 0.005, Supplementary Figures 1C,D). In the GEPIA database, we identified that *ELN* was highly expressed in normal bladder samples (Supplementary Figure 2A), while *DSC2* was highly expressed in BLCA samples (Supplementary Figure 2B). We also found that the expression levels of both *ELN* and *DSC2* were significantly different when compared by disease stages (Supplementary Figures 2C,D). The results of survival analyses elucidated that the high expression level of *ELN* was associated with worse overall survival (OS) in BLCA patients, while there was no survival difference of *DSC2* (Supplementary Figures 2E,F). We then investigated the expression levels of *ELN* and *DSC2* in various malignancies in the UALCAN database (Supplementary Figures 3A,B). Consistent with the above results, we observed that *ELN* was primarily expressed in normal bladder samples (Supplementary Figure 4A), while *DSC2* was primarily expressed in BLCA samples (Supplementary Figure 4B). Meanwhile, we also identified that high expression levels of *ELN* and *DSC2* were correlated with reduced OS in patients with BLCA (Supplementary Figures 4C,D). To explore the protein expression levels of *ELN* and *DSC2* in BLCA, we then exploited the HPA database to obtain the immunohistochemistry results of these genes. We identified that the protein expression levels of both *ELN* and *DSC2* were high in BLCA samples (Supplementary Figures 5A,B). Subsequently, the Kaplan–Meier Plotter database was utilized to confirm the survival difference of these genes in BLCA, suggesting that the high expression levels of *ELN* and *DSC2* were associated with poor OS in these individuals (Supplementary Figures 5C,D). Ultimately, the TIMER web server was used to investigate the prognostic value of *ELN*, *DSC2*, and the six subsets of immune cells in BLCA. We identified that the high expression levels of *ELN* and *DSC2* were also associated with worse OS in BLCA (Supplementary Figure 6A), which was compatible with the previous results. The multivariate Cox analysis showed that Macrophages, *DSC2*, and *ELN* were independent prognostic factors in BLCA (Supplementary Table 3). Besides, TargetScan was used to predict the binding sites of *hsa-miR-29c-3p* on *ELN* and *DSC2*. Not surprisingly, we found potential binding sites of *hsa-miR-29c-3p* on *ELN* (Supplementary Figure 6B) and *DSC2* (Supplementary Figure 6C).

## Discussion

Bladder cancer is responsible for 20% of cancer-related death worldwide (Klotz and Brausi, 2015). In spite of achievements in the screening, diagnosis, and treatment of BLCA have been made in recent decades, especially the clinical application of next-generation sequencing (NGS) technology and immunotherapy, it remains one of the most aggressive and lethal malignant types (Babjuk et al., 2017). Recently, numerous studies have highlighted that tumor-infiltrating immune cells and ceRNA networks were involved in the development, progression, and prognosis in various malignancies (Huang et al., 2019a, b, 2020). Nevertheless, there were no combined networks for predicting the prognosis in BLCA. Herein, we conducted the current study to investigate the prognostic value of immune cells and construct the ceRNA networks to predict the prognosis of BLCA patients using the TCGA dataset.

In the present study, we constructed the ceRNA network, which was composed of 7 lncRNAs, 15 miRNAs, and 110 mRNAs based on the interactions of 15 lncRNA-miRNA pairs and 154 miRNA-mRNA pairs by using the transcription profiles from TCGA BLCA cohort. In the ceRNA network, we observed that the expression levels of *ELN*, *SREBF1*, *DSC2*, *TTLL7*, *DIP2C*, *SATB1*, *hsa-miR-20a-5p*, and *hsa-miR-29c-3p* were significantly correlated with the OS in patients with BLCA. Ultimately, a nomogram was constructed to predict the survival probability of these individuals. According to the results of hypergeometric testing and correlation analysis, we identified that lncRNA *H19*, *hsa-miR-29c-3p*, *ELN*, and *DSC2* were significantly correlated in the network. Furthermore, we also found that *hsa-miR-29c-3p* was negatively correlated with neutrophils infiltration, *ELN* was negatively correlated with Tfh infiltration, and *DSC2* was positively correlated with neutrophils infiltration. Therefore, we inferred that *hsa-miR-29c-3p*, *ELN*, *DSC2*, neutrophils, and Tfh might play crucial roles in the progression of BLCA.

*miR-29c-3p*, which was reported as a tumor suppressor in the miRNAs family (Schmitt et al., 2013), plays a protective role in various malignancies, such as head and neck cancers (Liu et al., 2013; Hudcova et al., 2016; Fang et al., 2019), gastrointestinal cancers (Ding et al., 2011; Matsuo et al., 2013; Chen et al., 2017), hepatobiliary cancers (Wang et al., 2015; Shu et al., 2017), breast cancer (Bhardwaj et al., 2017), and BLCA (Inamoto et al., 2018). Numerous studies have shown that the low expression level of *miR-29c* was associated with worse tumor differentiation, advanced disease stage, and poor prognosis. Furthermore, it was reported that *miR-29c* inhibits the proliferation, invasion, and metastasis of tumors and promotes apoptosis via regulating various oncogenes, biological pathways, cell cycles, and epithelial to mesenchymal transition (EMT) (Han et al., 2015; Zhang et al., 2016; Li et al., 2017; Yu et al., 2017). *ELN* (also known as elastin) plays an essential role in elasticity properties in soft tissues, is a key member of the extracellular matrix (ECM) family (Salesse et al., 2018). Li et al. indicated that *ELN* regulates cancer cell adhesion, migration, and invasion by inducing the EMT process in colorectal cancer (Li et al., 2020). Salesse et al. (2018) also identified that *ELN* promotes human breast cancer cell invasiveness via increasing the activity of matrix metalloproteinases (MMPs), which is the main enzyme to cleave ECM products. Besides, Yasui et al. (2016) indicated that *ELN* could be served as a biomarker for the development of hepatocellular carcinoma. However, the role of *ELN* in BLCA has not been reported yet. We are the first reported the expression level of ELN in BLCA, and we identified that the high expression level of *ELN* was associated with poor prognosis in these individuals. *DSC2* is the most widespread and ubiquitous desmosome isoform, encoding desmocollin 2 protein, which belongs to the cadherin family of calcium-dependent cell adhesion molecules (Dusek et al., 2007). Previously published studies suggested that *DSC2* is primarily expressed in gastrointestinal cancers and skin squamous cell carcinoma (Khan et al., 2006; Anami et al., 2010). Recently, Hayashi et al. (2011) reported that *DSC2* is also expressed in UCC, and it serves as a new immunohistochemical marker indicative of squamous differentiation in UCC. Furthermore, they found that high expression level of *DSC2* was correlated with advanced disease stage and decreased OS in UCC patients (Hayashi et al., 2011). However, there was no study explained the underlying mechanisms at present.

We also investigated the prognostic significance of immune cells in BLCA using the CIBERSORT algorithm, indicating that T cells CD8, Tfh, and neutrophils were referred to as independent prognostic factors in BLCA. Meanwhile, the co-expression analysis showed that *ELN* was negatively correlated with Tfh infiltration, while *DSC2* was positively correlated with neutrophils infiltration. Tfh are a subset of CD4+ T cells specialized to regulate antibody responses by regulating the clonal selection of germinal center B cells and generating antibody signals (Eivazi et al., 2016). Mounting evidence suggested that Tfh play crucial roles in long-lived humoral immunity. Dysregulation in Tfh cell generation has been implicated in various diseases, such as autoimmune diseases, immunodeficiency, cancer, asthma, and other allergic diseases. Numerous studies have shown that Tfh cell infiltration in the tumor was positively correlated with survival, including non-small cell lung cancer, breast cancer, prostate cancer, colorectal cancer and so on (Gu-Trantien et al., 2013; Tan et al., 2015; Ma et al., 2016; Shi et al., 2018). Li et al. (2014) indicated that Tfh promotes the effector functions of CD8+ T cells via the provision of IL-21, which is downregulated due to PD-1/PD-L1-mediated suppression in colorectal cancer, thus leading better survival in these patients (Shi et al., 2018). Tumor-infiltrating neutrophils (TIN) play dual roles in the tumor biological process as an important inflammation component. TIN can be polarized into either an anti-tumoral (N1) or a pro-tumoral (N2) phenotype and play different functions (Fridlender et al., 2009). Previous studies indicated that the N1 phenotype TIN plays an anti-tumoral role by inducing cytotoxicity, mediating tumor rejection, and anti-tumoral immune memory (Liu et al., 2018). On the contrary, the N2 phenotype TIN plays a pro-carcinogenic effect by promoting angiogenesis, invasion, metastasis, and immunosuppression (Liu et al., 2018). According to the previously published studies, it showed that TIN play pro-carcinogenic effect and are correlated with worse prognosis in the majority of malignancies, such as esophageal carcinoma, non-small cell lung cancer, and renal cancer (Jensen et al., 2009; Wang et al., 2014). Recently, Liu et al. (2018) revealed that elevated TIN was associated with poor OS of BLCA patients, and it can be served as an independent unfavorable prognostic marker in BLCA.

Taken together, we inferred that *hsa-miR-29c-3p*, *ELN*, *DSC2*, Tfh, and neutrophils might play crucial roles in the progression of BLCA. Although the result is meaningful and exciting, there are no relative biological experiments to support our findings. We will continue to explore the potential mechanism of how the *hsa-miR-29c-3p* regulates the expression of *ELN* and *DSC2* and affects the infiltration of the immune cells in BLCA in our future work. To the best of our knowledge, this is the first study that investigated the prognostic significance of immune cells and constructed ceRNA networks in BLCA. Besides, our study constructed two nomograms that can accurately predict the survival probability of BLCA patients. Our study also inferred that the mechanism of *hsa-miR-29c-3p* regulates the expression of *ELN* and *DSC2*, and the infiltration of Tfh and neutrophils might play pivotal roles in the progression of BLCA. We believe that our study will provide a prospective insight into this field. However, despite the advantages of this study, there are also several inevitable limitations in the present study. First, there was no relevant basic experiment to detect the expression levels of the identified key members of ceRNA networks in cell lines or clinical samples; Second, although we inferred that *hsa-miR-29c-3p*, *ELN*, *DSC2*, Tfh, and neutrophils might play crucial roles in the progression of BLCA, we did not further explore the potential mechanisms that how the *hsa-miR-29c-3p* regulate the expression of *ELN* and *DSC2* and affect the infiltration of the immune cells. Last but not least, although using a combined network analysis to predict the survival outcomes of BLCA patients is novel in this field. Considering the results are concluded according to the correlation analysis and there are no relevant biological experiments. Therefore, the effectiveness of this method needs to be further validated.

## Conclusion

In summary, our study provided a systematic analysis of ceRNA networks and tumor-infiltrating immune cells in BLCA patients and constructed two nomograms to predict the survival probability in these individuals. The results suggest that the mechanism of *hsa-miR-29c-3p* regulates the expression of *ELN* and *DSC2*, and the infiltration of Tfh and neutrophils might play pivotal roles in the progression of BLCA.

## Data Availability Statement

Publicly available datasets were analyzed in this study. This data can be found here: https://portal.gdc.cancer.gov.

## Author Contributions

XL, YY, TT, and ZR: conception and design. AJ, NL, and SB: provision of study material. AJ, JW, HG, XQZ, and XF: collection and/or assembly of data. AJ, NL, SB, MR, and XNZ: data analysis and interpretation. AJ, NL, and SB: manuscript writing. AJ, XL, and YY: final approval of the manuscript. All authors read and approved the final manuscript and agree to be accountable for all aspects of the research in ensuring that the accuracy or integrity of any part of the work are appropriately investigated and resolved.

## Conflict of Interest

The authors declare that the research was conducted in the absence of any commercial or financial relationships that could be construed as a potential conflict of interest.

## Footnotes

1 https://tcga-data.nci.nih.gov/tcga/

2 http://starbase.sysu.edu.cn/

3 https://www.oncomine.org/resource/main.html

4 http://gepia.cancer-pku.cn/

5 http://www.kmplot.com/

6 http://ualcan.path.uab.edu/cgi-bin/ualcan-res.pl

7 https://www.proteinatlas.org/

8 https://cistrome.shinyapps.io/timer/

9 http://www.targetscan.org/vert_72/

10 https://www.bioconductor.org/

## Supplementary Material

Supplementary Figure 1 Validation of *Eln* **(A,B)** and *Dsc2* **(C,D)** in multiple cancer types and multiple studies using the Oncomine database.

Supplementary Figure 2 Validation of *Eln* **(A,C,E)** and *Dsc2* **(B,D,F)** in Blca using the Gepia database.

Supplementary Figure 3 Validation of *Eln* **(A)** and *Dsc2* **(B)** in multiple cancer types using the Ualcan database.

Supplementary Figure 4 Validation of *Eln* **(A,C)** and *Dsc2* **(B,D)** in Blca using the Ualcan database.

Supplementary Figure 5 Validation of *Eln* and *Dsc2* in Blca using the Hpa database **(A,B)** and Kaplan–Meier Plotter database **(C,D)**.

Supplementary Figure 6 Validation of *Eln* and *Dsc2* in Blca using the Timer database **(A)**; the binding sites of *hsa*-miR-29c-3p on *Eln* **(B)** and *Dsc2* **(C)**.
