# Supplementary material for: The Construction and Analysis of Tumor-Infiltrating Immune Cells and ceRNA Networks in Bladder Cancer
Source: Front Genet. 2020 Dec 18;11:605767. doi: 10.3389/fgene.2020.605767 (PMC7775311; doi:10.3389/fgene.2020.605767)
Supplement: Supplementary Figure 1 — Validation of Eln (A,B) and Dsc2 (C,D) in multiple cancer types and multiple studies using the Oncomine database. [file Data_Sheet_1.PDF]

Figure S1

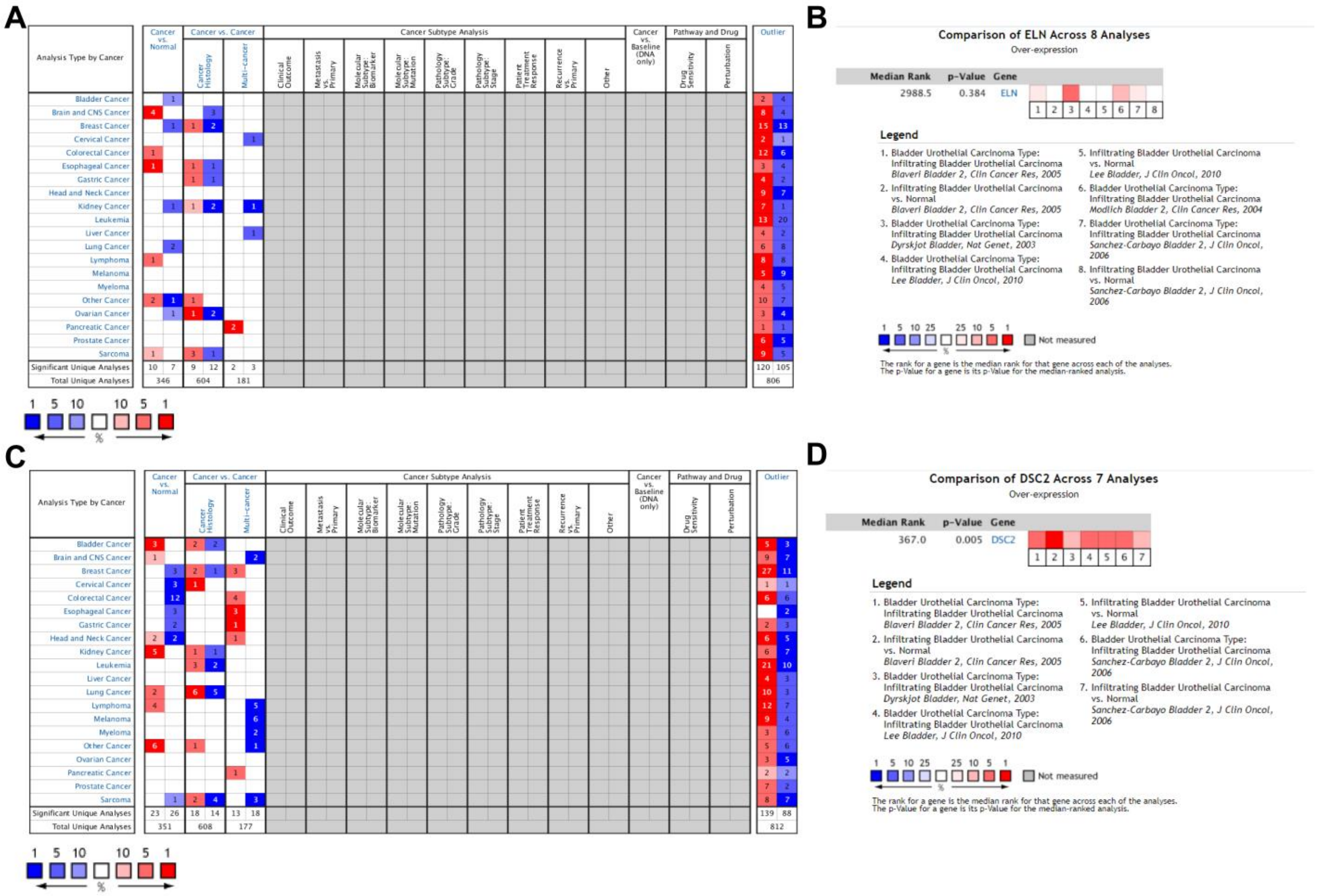
A
B
Comparison of ELN Across 8 Analyses
Over-expression
Median Rank p-Value Gene
2988.5 0.384 ELN
Legend
1. Bladder Urothelial Carcinoma Type: Infiltrating Bladder Urothelial Carcinoma Blaveri Bladder 2, Clin Cancer Res, 2005
2. Infiltrating Bladder Urothelial Carcinoma vs. Normal Blaveri Bladder 2, Clin Cancer Res, 2005
3. Bladder Urothelial Carcinoma Type: Infiltrating Bladder Urothelial Carcinoma Dyrskjot Bladder, Nat Genet, 2003
4. Bladder Urothelial Carcinoma Type: Infiltrating Bladder Urothelial Carcinoma Lee Bladder, J Clin Oncol, 2010
5. Infiltrating Bladder Urothelial Carcinoma vs. Normal Lee Bladder, J Clin Oncol, 2010
6. Bladder Urothelial Carcinoma Type: Infiltrating Bladder Urothelial Carcinoma Modlich Bladder 2, Clin Cancer Res, 2004
7. Bladder Urothelial Carcinoma Type: Infiltrating Bladder Urothelial Carcinoma Sanchez-Carbayo Bladder 2, J Clin Oncol, 2006
8. Infiltrating Bladder Urothelial Carcinoma vs. Normal Sanchez-Carbayo Bladder 2, J Clin Oncol, 2006
Not measured
The rank for a gene is the median rank for that gene across each of the analyses.
The p-Value for a gene is its p-Value for the median-ranked analysis.
C
D
Comparison of DSC2 Across 7 Analyses
Over-expression
Median Rank p-Value Gene
367.0 0.005 DSC2
Legend
1. Bladder Urothelial Carcinoma Type: Infiltrating Bladder Urothelial Carcinoma Blaveri Bladder 2, Clin Cancer Res, 2005
2. Infiltrating Bladder Urothelial Carcinoma vs. Normal Blaveri Bladder 2, Clin Cancer Res, 2005
3. Bladder Urothelial Carcinoma Type: Infiltrating Bladder Urothelial Carcinoma Dyrskjot Bladder, Nat Genet, 2003
4. Bladder Urothelial Carcinoma Type: Infiltrating Bladder Urothelial Carcinoma Lee Bladder, J Clin Oncol, 2010
5. Infiltrating Bladder Urothelial Carcinoma vs. Normal Lee Bladder, J Clin Oncol, 2010
6. Bladder Urothelial Carcinoma Type: Infiltrating Bladder Urothelial Carcinoma Sanchez-Carbayo Bladder 2, J Clin Oncol, 2006
7. Infiltrating Bladder Urothelial Carcinoma vs. Normal Sanchez-Carbayo Bladder 2, J Clin Oncol, 2006
Not measured
The rank for a gene is the median rank for that gene across each of the analyses.
The p-Value for a gene is its p-Value for the median-ranked analysis.

Figure S2

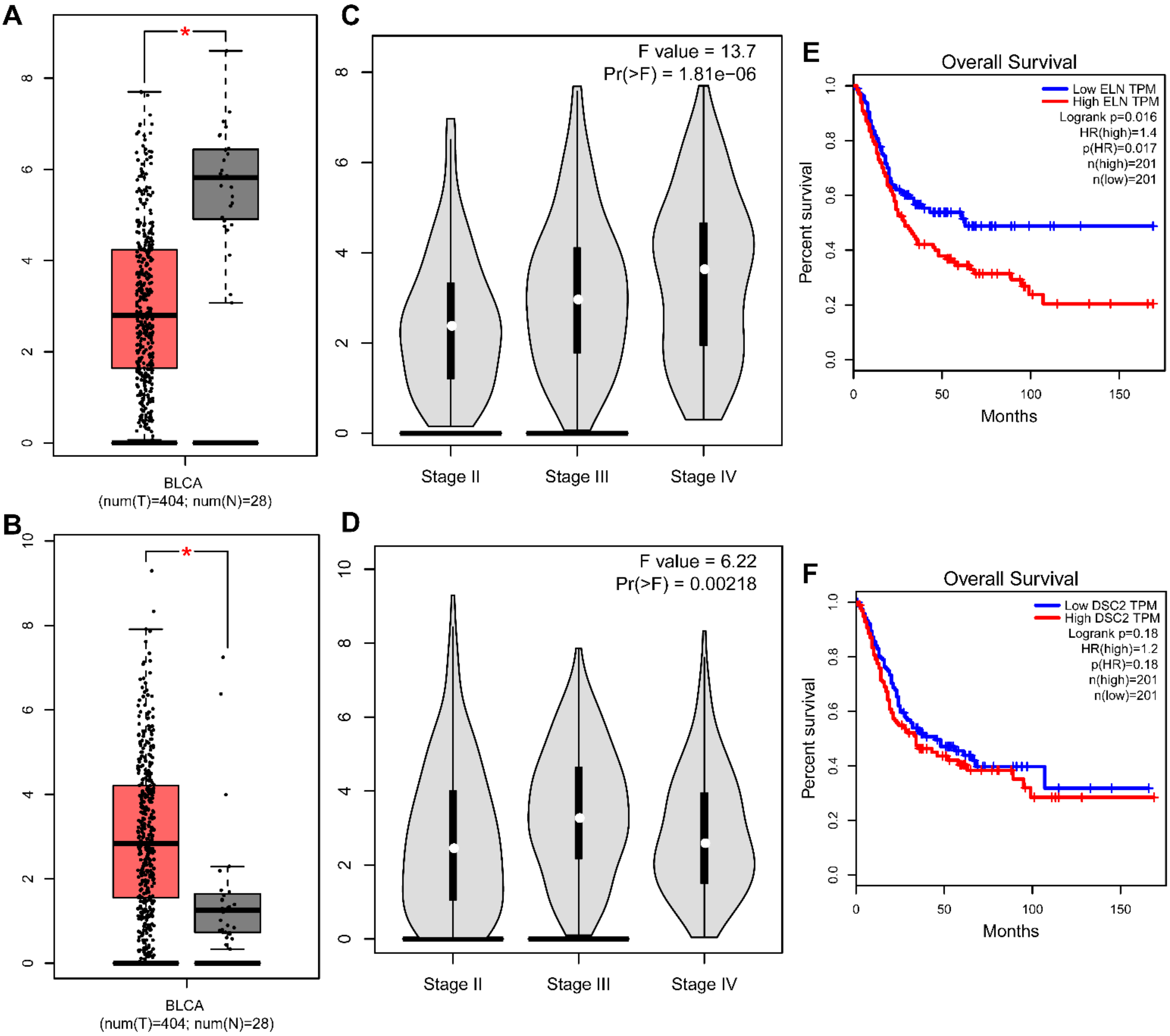
A
B
C
D
E
F
BLCA
(num(T)=404; num(N)=28)
F value = 13.7
Pr(>F) = 1.81e−06
F value = 6.22
Pr(>F) = 0.00218
Stage II
Stage III
Stage IV
Overall Survival
Low ELN TPM
High ELN TPM
Logrank p=0.016
HR(high)=1.4
p(HR)=0.017
n(high)=201
n(low)=201
Low DSC2 TPM
High DSC2 TPM
Logrank p=0.18
HR(high)=1.2
p(HR)=0.18
n(high)=201
n(low)=201
Percent survival
Months

Figure S3

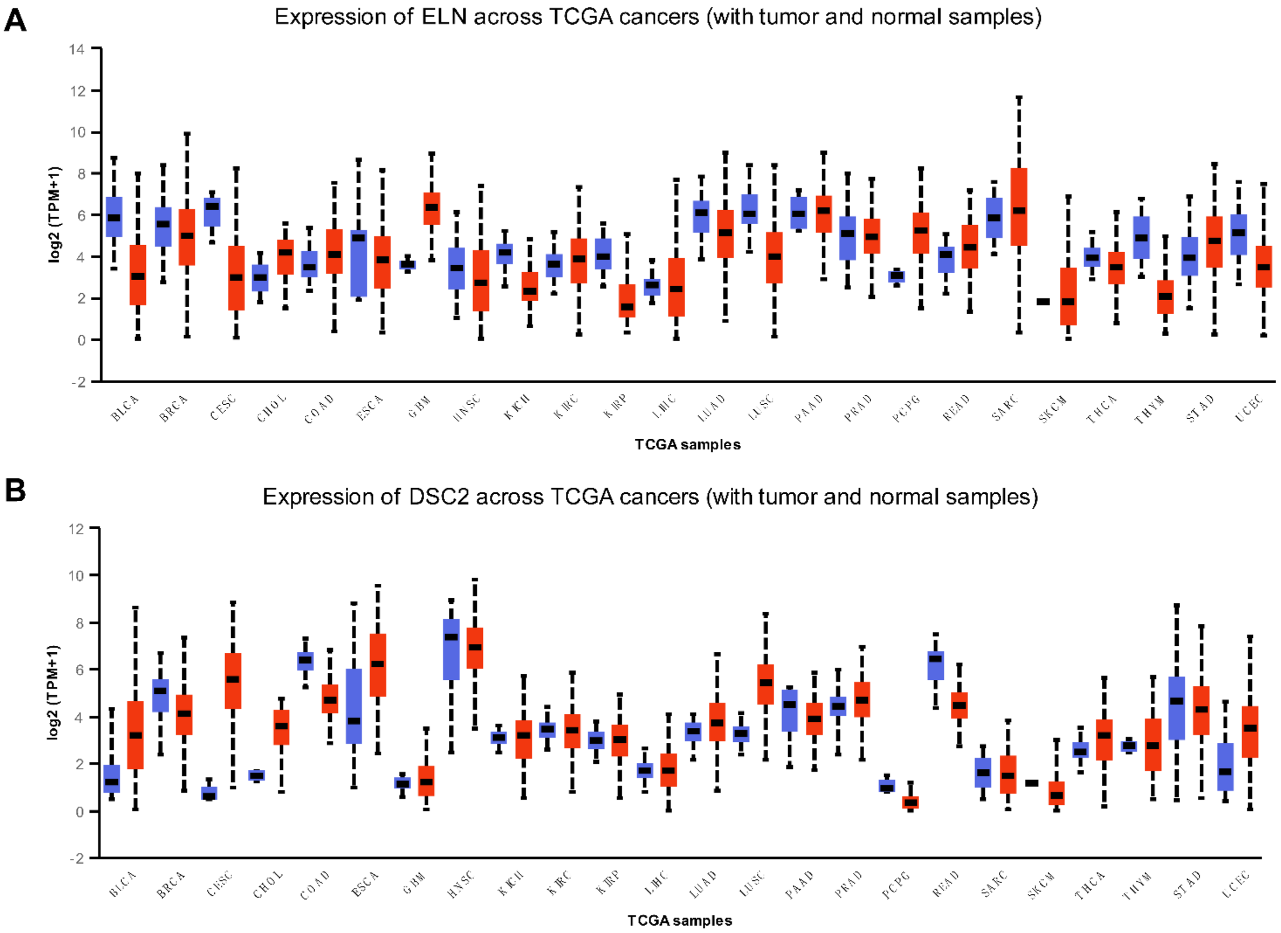
A
Expression of ELN across TCGA cancers (with tumor and normal samples)
log2 (TPM+1)
14
12
10
8
6
4
2
0
-2
BLCA
BRCA
CESC
CHOL
COAD
ESCA
GBM
HNSC
KICH
KIRC
KIRP
LIHC
LUAD
LUSC
PAAD
PRAD
PCPG
READ
SARC
SKCM
THCA
THYM
STAD
UCEC
TCGA samples
B
Expression of DSC2 across TCGA cancers (with tumor and normal samples)
log2 (TPM+1)
12
10
8
6
4
2
0
-2
BLCA
BRCA
CESC
CHOL
COAD
ESCA
GBM
HNSC
KICH
KIRC
KIRP
LIHC
LUAD
LUSC
PAAD
PRAD
PCPG
READ
SARC
SKCM
THCA
THYM
STAD
UCEC
TCGA samples

Figure S4

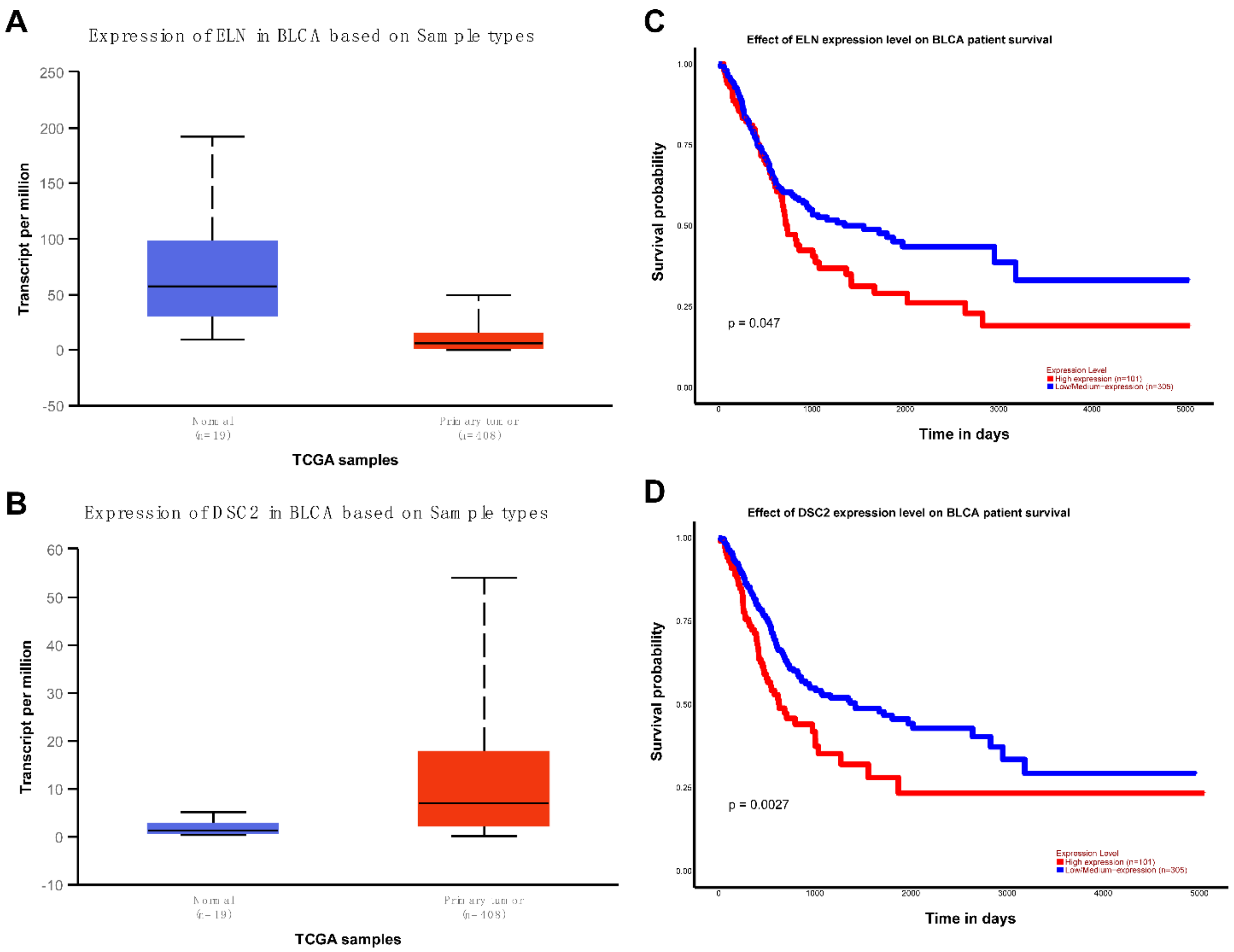
A
Expression of ELN in BLCA based on Sample types
Transcript per million
250
200
150
100
50
0
-50
Normal
(n=19)
Primary tumor
(n=408)
TCGA samples
B
Expression of DSC2 in BLCA based on Sample types
Transcript per million
60
50
40
30
20
10
0
-10
Normal
(n=19)
Primary tumor
(n=408)
TCGA samples
C
Effect of ELN expression level on BLCA patient survival
Survival probability
1.00
0.75
0.50
0.25
0.00
p = 0.047
Expression Level
High expression (n=101)
Low/Medium−expression (n=305)
0
1000
2000
3000
4000
5000
Time in days
D
Effect of DSC2 expression level on BLCA patient survival
Survival probability
1.00
0.75
0.50
0.25
0.00
p = 0.0027
Expression Level
High expression (n=101)
Low/Medium−expression (n=305)
0
1000
2000
3000
4000
5000
Time in days

Figure S5

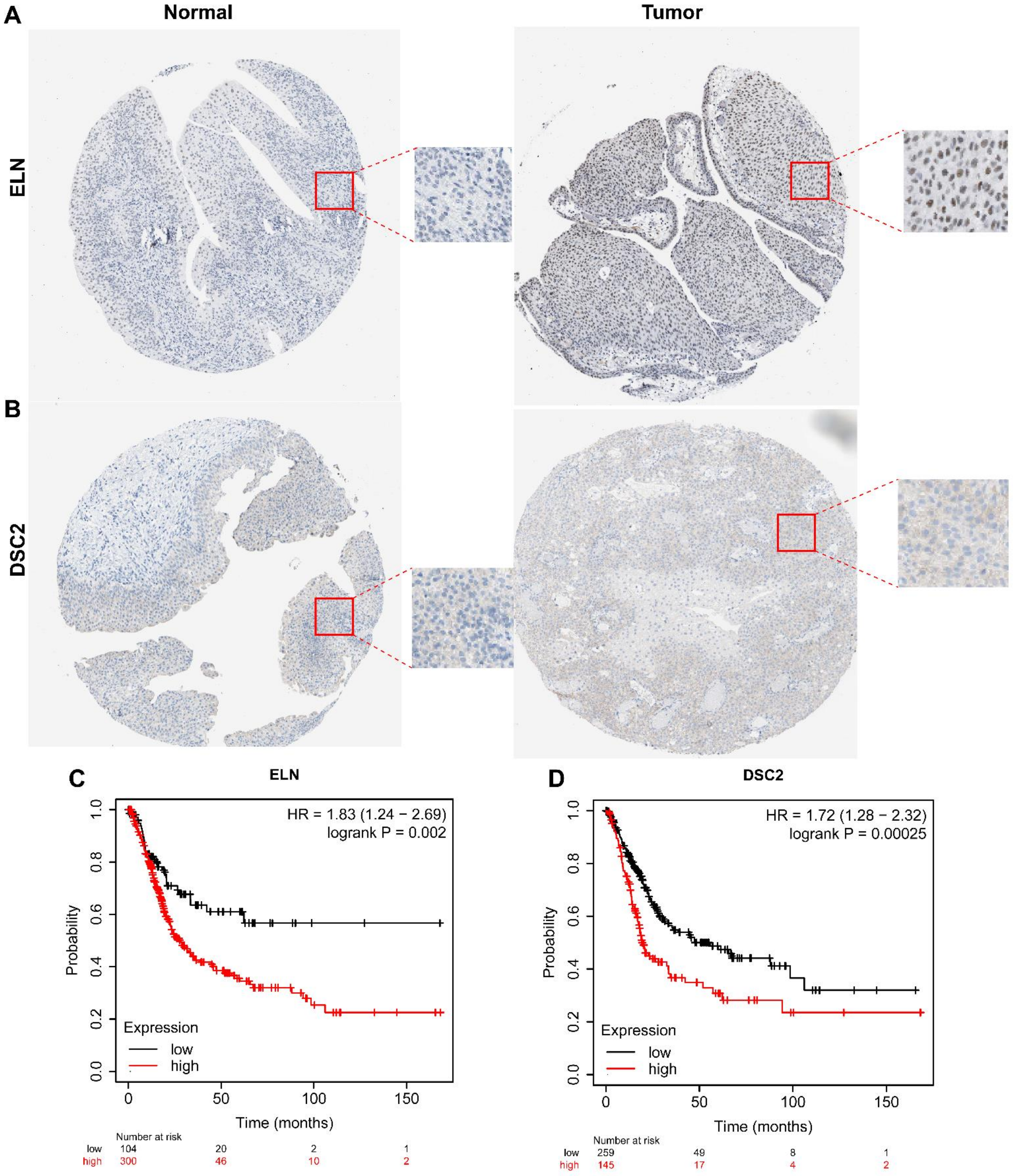

A
Normal
Tumor
ELN
B
DSC2
C
ELN
HR = 1.83 (1.24 − 2.69)
logrank P = 0.002
Probability
Time (months)
Expression
low
high
Number at risk
low 104 20 2 1
high 300 46 10 2
D
DSC2
HR = 1.72 (1.28 − 2.32)
logrank P = 0.00025
Probability
Time (months)
Expression
low
high
Number at risk
low 259 49 8 1
high 145 17 4 2

Figure S6

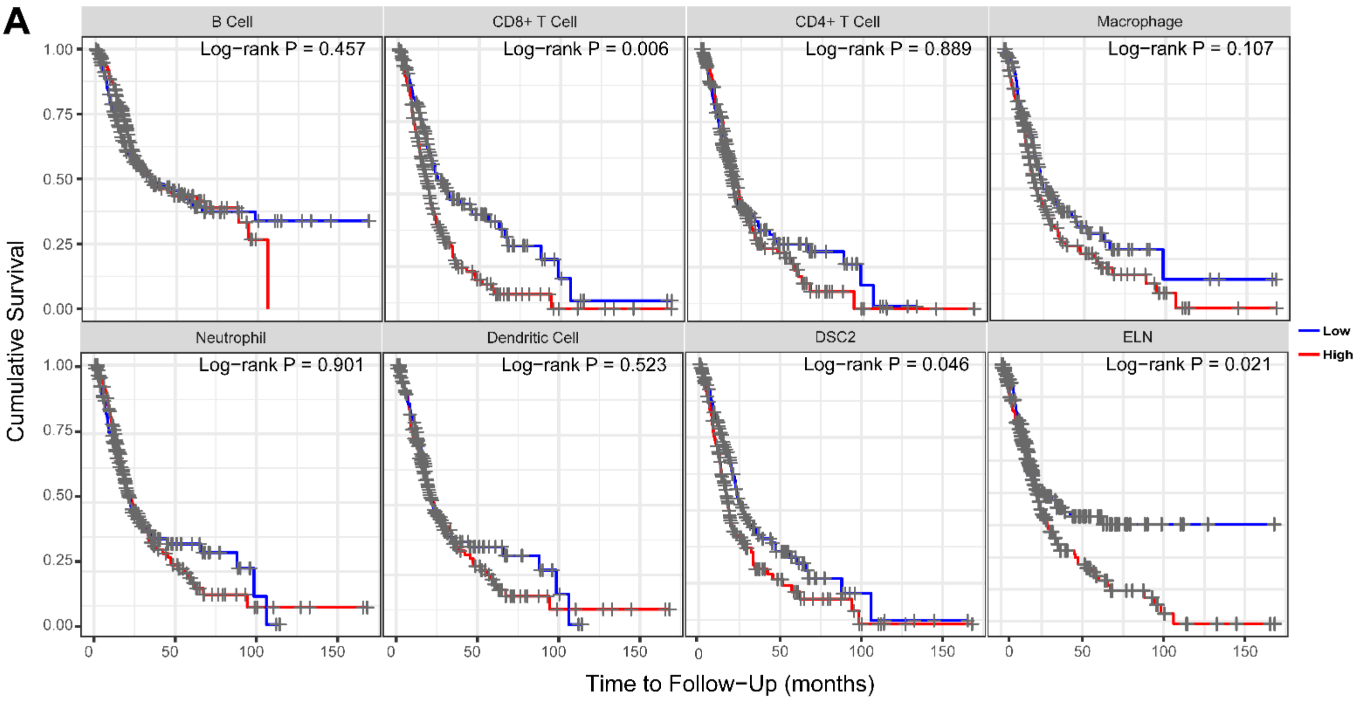

B

Binding Site of hsa-miR-29c-3p on ELN:

Target: 5' -CGACCUCAUCAACGUUGGUGCUA- 3'

miRNA : 3' AUUGGCUAAAGUUUACCACGAU 5'

C

Binding Site of hsa-miR-29c-3p on DSC2:

Target: 5' -AAUUAAGUGUUCAUGUGGUGCUU- 3'

miRNA : 3' AUUGGCUAAAGUUUACCACGAU 5'

Table S1. Clinical characteristics of 412 BLCA cancer patients in TCGA database.

**Characteristics**	**N (%)**
Age (years)	68.1±10.6
Gender	
Male	304 (73.8)
Female	108 (26.2)
Stage	
I	2 (0.5)
II	131 (31.8)
III	141 (34.2)
IV	136 (33.0)
Unknown	2 (0.5)
Grade	
High	388 (94.2)
Low	21 (5.1)
Unknown	3 (0.7)
AJCC T stage	
0	1 (0.2)
1	3 (0.7)
2	120 (29.1)
3	196 (47.6)
4	59 (14.3)
Unknown	33 (8.0)
AJCC N stage	
0	239 (58.0)
1	47 (11.4)
2	76 (18.4)
3	8 (1.9)
Unknown	42 (10.2)
AJCC M stage	
0	196 (47.6)
1	11 (2.7)
Unknown	205 (49.8)
Status	
Dead	180 (43.7)
Alive	232 (56.3)

Abbreviations: BLCA, bladder cancer; TCGA, The Cancer Genome Atlas; AJCC, American Joint Committee on Cancer.

Table S2. Hypergeometric testing and correlation analysis results of ceRNAs network.

lncRNAs	mRNAs	miRNAs	Correlation *P* Value	Hypergeometric test *P* Value
AC093010.3	LPP	hsa-miR-18a-5p	4.37E-30	4.30E-02
AC093010.3	MEF2D	hsa-miR-18a-5p	4.78E-21	4.94E-03
AC093010.3	PDE4D	hsa-miR-18a-5p	9.63E-16	5.59E-03
AC093010.3	ZBTB4	hsa-miR-18a-5p	1.27E-12	4.94E-03
AC093010.3	KLHL15	hsa-miR-18a-5p	4.04E-09	2.62E-02
AC093010.3	ZCCHC24	hsa-miR-18a-5p	7.08E-33	3.61E-02
AC093010.3	ZBTB47	hsa-miR-18a-5p	2.81E-27	1.56E-03
AC093010.3	FBXO31	hsa-miR-18a-5p	7.90E-09	2.78E-03
AC093010.3	LRRFIP1	hsa-miR-18a-5p	2.90E-04	6.93E-03
AC093010.3	THBS1	hsa-miR-18a-5p	5.90E-10	1.28E-03
AC093010.3	CTGF	hsa-miR-18a-5p	7.99E-18	5.78E-04
AC093010.3	CD302	hsa-miR-18a-5p	2.54E-19	1.08E-02
AC093010.3	DAAM2	hsa-miR-18a-5p	1.39E-26	7.79E-04
AC093010.3	KIAA0513	hsa-miR-18a-5p	1.03E-09	2.78E-03
AC093010.3	ATXN1	hsa-miR-18a-5p	3.47E-09	4.34E-02
AC093010.3	CNTNAP1	hsa-miR-18a-5p	3.68E-35	9.10E-03
AC093010.3	TNFAIP3	hsa-miR-18a-5p	1.12E-03	2.82E-03
AC093010.3	SATB1	hsa-miR-18a-5p	2.56E-13	2.41E-03
AC093010.3	LIF	hsa-miR-18a-5p	2.55E-07	4.06E-02
AC093010.3	KIT	hsa-miR-18a-5p	7.73E-17	1.73E-02
MAGI2-AS3	MEF2D	hsa-miR-374b-5p	3.57E-27	2.95E-02
MAGI2-AS3	TNS1	hsa-miR-374b-5p	2.11E-79	1.06E-02
MAGI2-AS3	PLPP3	hsa-miR-374b-5p	1.35E-27	1.56E-02
MAGI2-AS3	MYLK	hsa-miR-374b-5p	1.62E-77	4.97E-03
MAGI2-AS3	ARID5B	hsa-miR-374b-5p	7.54E-21	4.48E-02
MAGI2-AS3	PDE4D	hsa-miR-374b-5p	1.14E-22	3.20E-02
MAGI2-AS3	DMD	hsa-miR-374b-5p	2.75E-42	1.84E-02
MAGI2-AS3	ZCCHC24	hsa-miR-374b-5p	3.83E-70	1.30E-02
MAGI2-AS3	NFIX	hsa-miR-374b-5p	2.33E-30	9.89E-03
MAGI2-AS3	PNRC1	hsa-miR-374b-5p	1.19E-18	8.50E-03
MAGI2-AS3	LATS2	hsa-miR-374b-5p	2.34E-36	3.08E-02
MAGI2-AS3	SETD7	hsa-miR-374b-5p	1.03E-12	3.08E-02
MAGI2-AS3	RUSC2	hsa-miR-374b-5p	2.88E-45	5.49E-04
MAGI2-AS3	MEIS1	hsa-miR-374b-5p	1.52E-18	4.97E-03
MAGI2-AS3	UGCG	hsa-miR-374b-5p	4.72E-05	2.62E-04
MAGI2-AS3	C14orf28	hsa-miR-374b-5p	4.11E-12	2.04E-02
MAGI2-AS3	JMY	hsa-miR-374b-5p	2.47E-04	7.85E-03
MAGI2-AS3	STARD13	hsa-miR-374b-5p	3.16E-59	1.84E-02
MAGI2-AS3	RECK	hsa-miR-374b-5p	6.63E-70	3.60E-02
MAGI2-AS3	DIP2C	hsa-miR-374b-5p	1.83E-12	1.22E-02
MAGI2-AS3	LRCH2	hsa-miR-374b-5p	6.90E-63	1.06E-02
MAGI2-AS3	CDC42EP3	hsa-miR-374b-5p	2.41E-14	1.44E-03
MAGI2-AS3	CYBRD1	hsa-miR-374b-5p	2.21E-53	1.30E-02
MAGI2-AS3	TTLL7	hsa-miR-374b-5p	3.72E-10	3.56E-03
MAGI2-AS3	ZEB2	hsa-miR-374b-5p	1.13E-86	2.95E-02

MAGI2-AS3	TMEM55A	hsa-miR-374b-5p	4.98E-27	6.62E-03
MAGI2-AS3	FAM46A	hsa-miR-374b-5p	4.36E-11	3.47E-02
MAGI2-AS3	DUSP8	hsa-miR-374b-5p	1.54E-16	2.75E-03
MAGI2-AS3	SEC23A	hsa-miR-374b-5p	1.69E-20	4.97E-03
MAGI2-AS3	DUSP19	hsa-miR-374b-5p	1.60E-08	2.62E-04
MAGI2-AS3	AKT3	hsa-miR-374b-5p	3.28E-51	1.65E-02
MAGI2-AS3	PDLIM5	hsa-miR-374b-5p	3.30E-18	1.65E-02
MAGI2-AS3	LPAR1	hsa-miR-374b-5p	1.89E-18	1.57E-04
MAGI2-AS3	PDE4B	hsa-miR-374b-5p	7.94E-53	1.06E-02
MAGI2-AS3	TACC1	hsa-miR-374b-5p	3.83E-13	2.83E-02
MAGI2-AS3	EPB41L2	hsa-miR-374b-5p	4.08E-49	2.38E-03
MAGI2-AS3	PRDM1	hsa-miR-374b-5p	8.10E-11	3.20E-02
MAGI2-AS3	ZSWIM6	hsa-miR-374b-5p	9.32E-04	2.59E-02
MAGI2-AS3	PITX2	hsa-miR-374b-5p	6.78E-15	7.32E-04
MAGI2-AS3	FOXO1	hsa-miR-374b-5p	1.79E-16	1.94E-02
MAGI2-AS3	STK38L	hsa-miR-374b-5p	4.70E-11	1.94E-02
MAGI2-AS3	GADD45A	hsa-miR-374b-5p	3.48E-02	1.18E-03
MAGI2-AS3	ATP8B2	hsa-miR-374b-5p	6.17E-65	2.38E-03
MAGI2-AS3	TLE4	hsa-miR-374b-5p	9.68E-10	3.08E-02
MAGI2-AS3	PRKCA	hsa-miR-374b-5p	2.31E-03	1.73E-03
MAGI2-AS3	PI15	hsa-miR-374b-5p	2.14E-24	5.49E-04
MAGI2-AS3	SATB1	hsa-miR-374b-5p	2.14E-02	1.84E-02
MAGI2-AS3	PCDH7	hsa-miR-374b-5p	3.41E-20	9.89E-03
MAGI2-AS3	MSX1	hsa-miR-374b-5p	5.34E-27	2.62E-04
MAGI2-AS3	SPRY2	hsa-miR-374b-5p	6.46E-08	6.04E-03
MAGI2-AS3	NTN1	hsa-miR-374b-5p	7.74E-24	2.75E-03
MAGI2-AS3	PMEPA1	hsa-miR-374b-5p	5.74E-15	6.04E-03
MAGI2-AS3	EDIL3	hsa-miR-374b-5p	3.52E-39	2.62E-04
MAGI2-AS3	RIPOR2	hsa-miR-374b-5p	3.11E-24	3.92E-04
MAGI2-AS3	HUNK	hsa-miR-374b-5p	6.73E-13	2.62E-04
MAGI2-AS3	ID4	hsa-miR-374b-5p	5.26E-06	1.56E-02
MAGI2-AS3	SYBU	hsa-miR-374b-5p	1.62E-06	7.85E-03
AC074117.1	IL22RA1	hsa-let-7c-5p	1.19E-02	1.55E-04
SNHG3	RCC1	hsa-miR-340-5p	9.28E-11	1.08E-02
SNHG3	PLK4	hsa-miR-340-5p	1.23E-03	3.61E-03
SNHG3	EFNA4	hsa-miR-340-5p	1.61E-03	2.17E-02
SNHG3	SREBF1	hsa-miR-340-5p	1.91E-04	3.61E-03
SNHG3	GPT2	hsa-miR-340-5p	1.66E-09	3.61E-02
SNHG3	IGFBP3	hsa-miR-340-5p	4.15E-02	4.69E-02
PVT1	PLAG1	hsa-miR-17-5p, hsa-miR-20a-5p, hsa-miR-93-5p, hsa-miR-106b-5p, hsa-miR-20b-5p, hsa-miR-106a-5p	2.62E-02	1.38E-04
H19	TPM1	hsa-miR-29c-3p, hsa-miR-29a-3p	5.27E-03	6.32E-03
H19	SH3D19	hsa-miR-454-3p, hsa-miR-130b-3p	3.51E-02	1.69E-03
H19	TTYH3	hsa-miR-454-3p, hsa-miR-130b-3p	3.59E-02	8.63E-04
H19	ZBTB47	hsa-miR-29c-3p, hsa-miR-130b-3p, hsa-miR-29a-3p	4.54E-02	7.65E-03
H19	GADD45B	hsa-miR-454-3p, hsa-miR-130b-3p	1.42E-03	5.97E-04
H19	RAI2	hsa-miR-454-3p, hsa-miR-130b-3p	3.75E-02	5.97E-04
H19	TSPAN18	hsa-miR-454-3p, hsa-miR-130b-3p	4.74E-04	1.10E-02
H19	CCDC137	hsa-miR-454-3p, hsa-miR-130b-3p	3.26E-02	2.11E-02

H19	ARHGAP1	hsa-miR-454-3p, hsa-miR-130b-3p	2.57E-03	8.63E-04
H19	JCAD	hsa-miR-454-3p, hsa-miR-130b-3p	2.95E-02	4.07E-02
H19	MAPRE2	hsa-miR-29c-3p, hsa-miR-29a-3p	2.79E-02	8.48E-03
H19	COL7A1	hsa-miR-29c-3p, hsa-miR-29a-3p	5.59E-05	1.17E-03
H19	SLC25A22	hsa-miR-29c-3p, hsa-miR-29a-3p	2.32E-03	1.40E-02
H19	SRPX	hsa-miR-454-3p, hsa-miR-130b-3p	3.85E-02	4.54E-03
H19	CNTNAP1		2.07E-02	2.54E-02
H19	ELN	hsa-miR-29c-3p, hsa-miR-29a-3p	2.51E-03	6.32E-03
H19	SH3RF3	hsa-miR-29c-3p, hsa-miR-29a-3p	3.56E-03	3.10E-03
H19	FSTL1	hsa-miR-29c-3p, hsa-miR-29a-3p	7.56E-04	3.51E-02
H19	VAV2	hsa-miR-454-3p, hsa-miR-130b-3p	1.52E-02	2.54E-02
H19	KBTBD8	hsa-miR-29c-3p, hsa-miR-454-3p, hsa-miR-130b-3p, hsa-miR-29a-3p	1.44E-02	3.95E-04
H19	PI15	hsa-miR-29c-3p, hsa-miR-29a-3p	4.94E-03	1.99E-03
H19	RFLNB	hsa-miR-29c-3p, hsa-miR-29a-3p	1.99E-02	4.54E-03
H19	RASD1	hsa-miR-454-3p, hsa-miR-130b-3p	4.65E-02	1.09E-03
H19	TIMP2	hsa-miR-454-3p, hsa-miR-130b-3p	6.69E-04	2.49E-03
H19	LRIG1	hsa-miR-454-3p, hsa-miR-130b-3p	3.94E-02	3.87E-02
H19	PMEPA1	hsa-miR-454-3p, hsa-miR-130b-3p	3.57E-05	1.09E-03
H19	PTHLH	hsa-miR-29c-3p, hsa-miR-29a-3p	5.55E-05	3.56E-03
H19	KIF26A	hsa-miR-29c-3p, hsa-miR-29a-3p	1.58E-03	1.99E-03
H19	EFEMP1	hsa-miR-29c-3p	1.04E-02	2.35E-02
H19	MMP2	hsa-miR-29c-3p, hsa-miR-29a-3p	2.04E-04	6.32E-03
H19	DSC2	hsa-miR-29c-3p, hsa-miR-29a-3p	2.05E-02	1.10E-02
MCF2L-AS1	ACYP1	hsa-miR-33a-5p	2.85E-03	3.61E-03
MCF2L-AS1	HOXC13	hsa-miR-33a-5p	1.44E-02	2.53E-02

Table S3. Multivariate Cox regression analysis of immune cells infiltration and related genes in of BLCA in TIMER database.

**Variables**	**Coefficient**	**HR**	**95%CI**		***P***
			Lower	Upper	
B cell	-2.83	0.06	0	1.07	0.055
CD8+ T cell	1.06	2.90	0.20	42.80	0.439
CD4+ T cell	-2.34	0.10	0	4.29	0.227
Macrophage	3.00	20.13	1.45	279.80	*
Neutrophil	-3.04	0.05	0	5.83	0.215
Dendritic	0.12	1.13	0.25	5.10	0.871
DSC2	0.17	1.19	1.09	1.30	***
ELN	0.13	1.13	1.02	1.26	*

Abbreviations: BLCA, bladder cancer; TIMER, Tumor IMmune Estimation Resource; HR, hazard ratio; CI, confidential interval.

R square =0.101 (max possible =9.91e-01); Likelihood ratio test *P*=1.39e-06; Wald test P=1.13e-06; Score (logrank) test *P*=9.99e-07.

* *P*<0.05, ** *P*<0.01, *** *P*<0.001.
